# Precision of genetic parameters and breeding values estimated in marker assisted BLUP genetic evaluation

**DOI:** 10.1186/1297-9686-41-26

**Published:** 2009-03-04

**Authors:** Stefan Neuner, Christian Edel, Reiner Emmerling, Georg Thaller, Kay-Uwe Götz

**Affiliations:** 1 Bavarian State Research Center for Agriculture, Institute of Animal Breeding, D-85580 Grub, Germany; 2 Christian-Albrechts-University, Institute of Animal Breeding and Husbandry, D-24098 Kiel, Germany

## Abstract

In practical implementations of marker-assisted selection economic and logistic restrictions frequently lead to incomplete genotypic data for the animals of interest. This may result in bias and larger standard errors of the estimated parameters and, as a consequence, reduce the benefits of applying marker-assisted selection. Our study examines the impact of the following factors: phenotypic information, depth of pedigree, and missing genotypes in the application of marker-assisted selection. Stochastic simulations were conducted to generate a typical dairy cattle population. Genetic parameters and breeding values were estimated using a two-step approach. First, pre-corrected phenotypes (daughter yield deviations (DYD) for bulls, yield deviations (YD) for cows) were calculated in polygenic animal models for the entire population. These estimated phenotypes were then used in marker assisted BLUP (MA-BLUP) evaluations where only the genotyped animals and their close relatives were included.

Models using YD of cows (bull dams) in addition to DYD of bulls resulted in much smaller standard errors for the estimated variance components. The bias in DYD models was larger than in models including YD. Depth of pedigree had the strongest impact on the standard errors of all the estimated variance components. As expected, estimation of variance components was less precise with larger proportions of animals without genotypes in the pedigree. Accuracies of MA-BLUP breeding values for young bull candidates were strongly affected by the inclusion of cow information, but only marginally influenced by pedigree depth and proportions of genotyped animals.

## Background

Advances in molecular genetics have led to the identification of several genes and of genetic markers linked or associated with genes that affect traits of interest in livestock (QTL). Once QTL are detected, the aim of animal breeders is to integrate linked markers for QTL into the breeding program, in so-called marker assisted selection schemes (MAS). An overview about different possibilities to apply marker- and gene-assisted selection is given by Dekkers [1]. While the highest benefits are expected from gene-assisted selection using direct markers [1], in many cases, practical applications of MAS have to rely on anonymous markers that are often assumed to be in population-wide linkage equilibrium with the QTL.

The statistical model for using marker information in BLUP (best linear unbiased prediction) (MA-BLUP) genetic evaluations was developed by Fernando and Grossman [2]. The MA-BLUP methodology allows the simultaneous estimation of QTL and polygenic effects. The QTL effect is accounted for in the mixed model as an extra random effect with the covariance structure proportional to the IBD (identity by descent) matrix at the QTL position given the linked markers [2].

Components of an integrated system to apply MAS for routine evaluations are given by Dekkers [1]. In nearly all implementations, it will be necessary to take three decisions: i) how many animals are to be included in the MA-BLUP model, ii) which phenotypes should be used, and iii) how much effort is justified in order to completely genotype ancestors of the current young bull generation.

The aim of our study is to examine these three questions with respect to bias and standard errors of estimated variance components and accuracies of MA-BLUP breeding values by means of simulation.

## Methods

A stochastic simulation model was applied to generate a data set that was then analyzed with five different models. Each simulation cycle consisted of two phases: data generation and analysis of the simulated data sets. The number of replicates for each variant was 100.

### Data generation

In the simulation, data was generated for a conventional dairy cattle breeding scheme on a small scale. The general procedure is described in detail by Neuner *et al*. [3]. Parameters of the simulated population and base parameters of the progeny-testing program are shown in Table 1. The time horizon for data generation was 34 years in the current study.

**Table 1:** Simulated characteristics of the cow population and of the breeding program

Cow population	
Milking cows, nb	20,000
Cows in lactation, %	%
Lactation 1	35
Lactation 2	27
Lactation 3	21
Lactation 4	17
Bull dams, nb	250
Age at first calving, months	24
Intergestation period, months	12

AI bull population	

Age at birth of first progeny, months	24
Age at first breeding value estimation, months	60
Service life as proven bull, months	48
Maximum age, months	108
Bulls sampled, nb/year	72
Sires for insemination service per year, nb	10
Sires of bulls used per year, nb	4
Daughter records per bull sampled, nb	70

A single-trait model for 305-day milk yield with a heritability of 0.36 and an additive genetic variance of 260,100 kg^2 ^was chosen. Genetic parameters were in agreement with the actual first lactation parameters of German Fleckvieh [4]. The overall breeding value of each animal was the sum of a 'residual polygenic breeding value' and a 'QTL breeding value'. A single biallelic QTL with an allele frequency of 0.5 was assumed and the QTL was bracketed by two marker loci located 3 cM and 2 cM apart, each with 10 alleles but different allelic distributions. Allele frequencies for the marker 3 cM apart from the QTL were 40, 19, 15, 12, 7, 2, 2, 1, 1 and 1% (polymorphic information content, PIC = 0.732), and for the marker 2 cM apart were 60, 20, 8, 4, 2, 2, 1, 1, 1 and 1% (PIC = 0.555), respectively. The Haldane mapping function [5] was assumed to simulate meiosis. The multipoint polymorphism content [6] for the simulated QTL position was 0.753.

All calculations assumed a QTL accounting for 20% of the overall additive genetic variance of the trait investigated, without dominance effects at the QTL.

### Analysis of simulated data sets

In routine genetic evaluations of dairy cattle all pedigreed animals are included. However, when applying MAS, only a small fraction of animals might be genotyped at genetic markers. Since only the genotyped animals provide information for the estimation of QTL variance components and breeding values in MA-BLUP models, the 'two-step approach' as described by Liu *et al*. [7], Druet *et al*. [8] and Bennewitz *et al*. [9] was used in this study.

#### MA-BLUP using a two-step approach

In the first step, a classical polygenic animal model (AM) evaluation, assuming the true variance components are known, was conducted for the entire population in order to estimate daughter yield deviations (DYD) for bulls and yield deviations (YD) for cows, respectively [10]. The pedigree contained about 260,000 animals of which 251,000 were cows with phenotypic records. To estimate AM-based breeding values, DYD, and YD, the package MiX99 [11] was used.

The second evaluation step was applied only to a subset of genotyped animals from the population (MA-BLUP pedigree). Usually the pedigree used for MA-BLUP evaluations contains only young bull candidates, young bulls currently used, waiting bulls and their parents and one or more generations of ancestors. For the current study it was assumed that complete marker information was theoretically available for all animals in the MA-BLUP pedigree. Phenotypic observations in step 2 were either DYD of bulls alone, or DYD together with YD of cows. The different amount of information available for DYD was accounted for by applying weighting factors to DYD. YD were not weighted, because each cow had only one record in the current study (Druet 2006; personal communication) and fixed effects that may have an impact on the accuracy of the estimation of YD, *e.g*. herd effects, were not assumed. When using DYD and YD together in one model for MA-BLUP evaluations, it is necessary to consider that these two information types represent different amounts of genetic and residual variance. Neuner *et al*. [3] have provided a detailed description of this issue. In order to account for these differences weighting of information was necessary. The weighting factors EDC (effective daughter contributions, Fikse and Banos [12]) and **γ **[3] were applied to twice the DYD in DYD-YD evaluations.

An MA-BLUP model equivalent to that of Fernando and Grossman [2] was used for the estimation of MA-BLUP breeding values:

(1) *y*_*i *_= *μ *+ *u*_*i *_+ *v*_*i *_+ *e*_*i*_

where *y*_*i *_is the record (YD for dams and twice the DYD for sires) of individual *i*, *u*_*i *_is the residual polygenic effect of individual *i*, *v*_*i *_is the effect of QTL-genotype of individual *i *and *e*_*i *_is the residual. QTL effects were included in the evaluations in terms of the IBD (identical by descent) matrix. In contrast to Fernando and Grossman [2], IBD matrices applied to (1) are genotypic relationship matrices at the QTL and not gametic relationship matrices. According to (1) the total estimated breeding value (EBV) in MA-BLUP models is the sum of the estimates of the polygenic and the QTL effect obtained by solving the mixed model equations:

(2) $$EBV_{i}={\hat{u}}_{i}+{\hat{v}}_{i}$$

#### Pedigree for MA-BLUP

Based on the complete pedigree used for AM evaluations in step 1, two pedigrees were derived for MA-BLUP. The difference between them was the depth of the pedigree. The 'short pedigree' includes actual selection candidates for progeny testing and young bulls progeny tested during the last four years. In addition, all their parents and grandparents were included. Animals in the deep pedigree were actual selection candidates for progeny testing and bulls tested during the last eight years as well as parents and grandparents of all these bulls. In total, the short pedigree spanned three generations and contained 1,821 animals, whereas the deep pedigree comprised four generations with 2,671 animals. The main characteristics for these two pedigrees are given in Table 2. Table 2 is based on the observed numbers of animals during the simulation process.

**Table 2:** Main characteristics of the short and the deep pedigree and the related phenotypic data that are used for MA-BLUP evaluations

	Short pedigree	Deep pedigree
Animals, nb	1,821	2,671
Bulls, nb	790	1,071
Cows, nb	1,031	1,600
Animals with records (DYD, YD) in the MA-BLUP pedigree, nb	1,461	2,311
Bulls with record (DYD) in the MA-BLUP pedigree, nb	430	711
Cows with record (YD) in the MA-BLUP pedigree, nb	1,031	1,600
Waiting bulls without records in the MA-BLUP pedigree, nb	144	144
Young bull candidates without records in the MA-BLUP pedigree, nb	216	216

#### Schemes with missing genotypes

For random QTL models according to Fernando and Grossman [2] an IBD matrix reflects the covariance structure of QTL gametic effects in the pedigree. George *et al*. [13] have reported an extensive overview on algorithms to calculate IBD matrices even for complex pedigrees and incomplete marker information. They concluded that simulation-based algorithms like the multiple-site segregation sampler LOKI [14] are efficient tools to calculate IBD matrices for complex pedigree structures.

To analyze the effect of missing genotypes, three IBD matrices were calculated for each simulated data set and pedigree size. The first IBD matrix was calculated for the situation where all animals in the MA-BLUP pedigree were genotyped at all markers. Another two IBD matrices were built up for two different genotyping structures: moderate and extensive gaps. To generate the data sets with missing genotypes, realistic constraints were introduced. Old animals at the top of the pedigree are more often not genotyped than animals at the bottom of the pedigree, and missing genotypes occur more often for females than for males. Hence, missing genotypes were generated dependent on the position of an animal in the pedigree and its sex. Simulated proportions of missing genotypes for the two scenarios of incomplete genotyping are given in Table 3. Scenario 1 could be regarded as a situation that occurs at the start of MA-BLUP in realistic breeding programs. Genotypes are available for most bulls, but most of the older females in the pedigree are not genotyped. Scenario 2 is an extreme situation, with only very sparse genotypic information for the ancestors of current young bull candidates and progeny tested bulls of the last four (eight) years. Missing genotypes in scenario 2 depend on the missing genotypes in scenario 1, *i.e*. all animals having missing genotypes in scenario 1 have missing genotypes in scenario 2 as well.

**Table 3:** Proportions of genotypes that are assumed to be missing for moderate and extensive gaps in the genotyping structure

Missing genotypes	Sire	Paternal grandsire	Maternal grandsire	Dam	Paternal granddam	Maternal granddam
Scenario 1, moderate	0.15	0.30	0.50	0.30	0.85	0.90
Scenario 2, extensive	0.50	0.70	0.80	0.90	0.90	0.90

According to the position of an animal in the pedigree, probabilities for being non-genotyped were chosen.

In total, the proportion of missing genotypes is about 41% for scenario 1 and 61% for scenario 2 for both pedigree depths. All IBD matrices applied for MA-BLUP evaluations were genotypic relationship matrices for the QTL and calculated using the package LOKI [14].

Genetic parameters for MA-BLUP models and MA-BLUP EBV were estimated with the ASREML package [15] using a MA-BLUP model equivalent to that of Fernando and Grossman [2] and assuming the QTL position is known from the mapping experiment.

#### Parameter combinations applied

Overall, we have examined twelve different models for MA-BLUP evaluations (two information types, two pedigree depths and three degrees of genotyping gaps). In order to limit the amount of variants presented in the paper, we decided to use a stepwise presentation. First, we will compare the DYD model and the DYD-YD model only in the case of complete information and short pedigree. Second, we will proceed with the comparison of short and deep pedigree for the DYD-YD model only and finally we will discuss the effects of missing genotypes in the context of the DYD-YD model with a deep pedigree. The variants are summarized in the first three columns of Table 4.

**Table 4:** Simulated and estimated parameters for the estimation of variance components when evaluation models were only based on daughter yield deviations (DYD) for bulls or DYD for bulls in combination with yield deviations (YD) for cows

Phenotypic information	Pedigree depth	Missing genotypes	${\hat{\sigma }}_{a}^{2}$	${\hat{\sigma }}_{e}^{2}$	${\hat{\sigma }}_{qtl}^{2}$	log *LR*	σ^qtl2σ^a2	*s.e*.(${\hat{\sigma }}_{a}^{2}$)	*s.e*.(${\hat{\sigma }}_{e}^{2}$)	*s.e*.(${\hat{\sigma }}_{qtl}^{2}$)
DYD	short	none	244,267	664,150	55,460	1.855	0.227	39,033	478,491	38,232
DYD-YD	short	none	259,493	459,265	52,924	2.539	0.204	20,033	31,899	31,892
DYD-YD	deep	none	260,677	458,426	50,293	7.284	0.193	16,100	25,221	19,392
DYD-YD	deep	moderate	260,738	458,418	50,462	6.400	0.194	16,109	25,246	20,996
DYD-YD	deep	extensive	260,899	458,271	51,020	5.373	0.196	16,133	25,284	23,333

*Simulated parameters*			*260,100*	*462,400*	*52,020*		*0.200*			

Different pedigree structures (short and deep) and levels of missing genotypes (none, moderate and extensive) were applied; parameters shown are the additive genetic variance (${\hat{\sigma }}_{a}^{2}$), the residual variance (${\hat{\sigma }}_{e}^{2}$), the genetic variance explained by one QTL (${\hat{\sigma }}_{qtl}^{2}$), the log likelihood ratio (log *LR*), the ratio of ${\hat{\sigma }}_{a}^{2}$ and ${\hat{\sigma }}_{qtl}^{2}$, and the estimated standard errors for the estimated variance components (*s.e*.); the values are averages over 100 replicates

### Parameters studied

Parameters considered for the estimation of variance components were the bias of estimated variance components and their standard errors.

The estimates deviation from the simulated parameters was used to check for bias due to the pedigree depth and/or missing genotypes. Standard errors of the estimates were used to assess the precision of estimates between different models. In order to assess the fit of the genetic model, the likelihood ratio test (*LRT *= -2 ln(*L*_0_(*no QTL present*)-*L*_1_(*QTL present*)) was calculated. *L*_0 _and *L*_1 _represent the likelihood values at the REML solutions of evaluations where no QTL was assumed to be segregating (*no QTL present*), and where a QTL was assumed to be segregating (*QTL present*), respectively.

To examine the impact of the different models on the estimation of MA-BLUP breeding values, the correlation of true and estimated breeding values was calculated for each group of animals (bulls, cows, young bull candidates).

## Results

Results presented are the averages of 100 replicates for a 20% simulated proportion of genetic variance explained by the QTL. Even if QTL variances close to zero were estimated in single replicates as an effect of a weak data structure, especially in DYD models for short pedigrees, these estimates were not excluded from the analysis in all investigated situations. This was necessary, as it was our interest, to elaborate and characterize the range of estimated parameters when a QTL is known to segregate with a fixed variance. In contrast, studies that aim at a possible gain due to MAS [16] allow for the exclusion of replicates that will not give benefits for the breeding program, because structure of the data and relevant parameters indicate that MAS will not improve selection.

### Variance component estimation

Results for the estimation of variance components for the investigated combinations of phenotypic information, pedigree depth and missing genotypes are summarized in Table 4. The first two lines of table 4 concern the question: which amount of phenotypic information should be used? In DYD models, the additive genetic variance (${\hat{\sigma }}_{a}^{2}$) was underestimated while the genetic variance explained by the QTL (${\hat{\sigma }}_{qtl}^{2}$) and the ratio of ${\hat{\sigma }}_{a}^{2}$ and ${\hat{\sigma }}_{qtl}^{2}$ were overestimated. The estimates in DYD-YD models were closer to the simulated parameters. The standard errors of the estimated variance components were lower in DYD-YD models.

**Figure 1:**
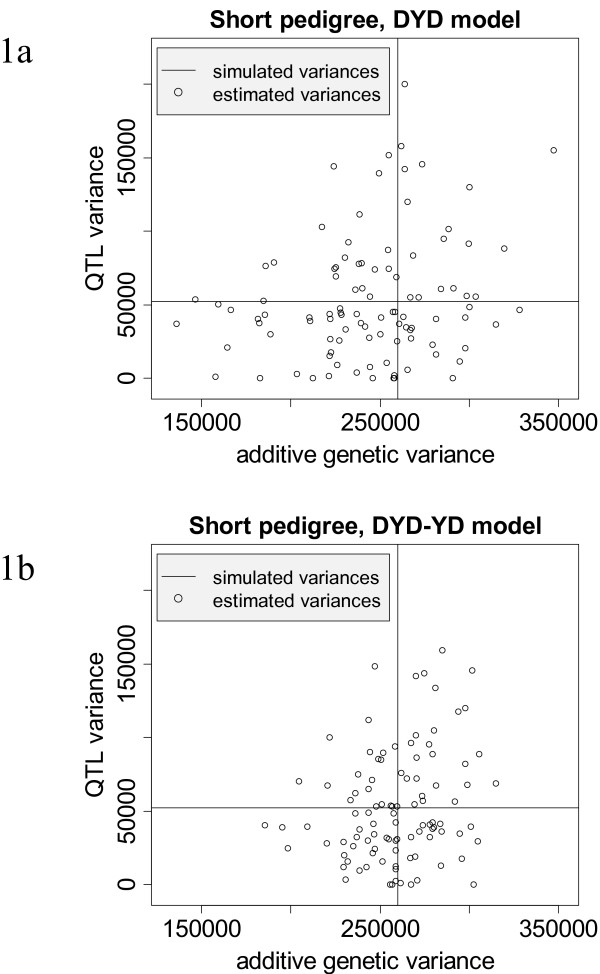
**Estimated (----) additive genetic variance and variance explained by a QTL compared to the simulated (----) parameters for a short depth of pedigree (see text for details)**. Figure 1a presents results for MA-BLUP models using only daughter yield deviations (DYD) of bulls as phenotypic information, whereas Figure 1b shows the results for MA-BLUP evaluations using DYD of bulls and yield deviations (YD) of cows together.

Figure 1 visualizes the variation of the estimated genetic variances in DYD and DYD-YD models. The amplitude in DYD-YD models is smaller for both ${\hat{\sigma }}_{a}^{2}$ and ${\hat{\sigma }}_{qtl}^{2}$. The benefit of using YD in addition to DYD is also confirmed by the higher LRT of the DYD-YD model (see Figure 1).

**Figure 2:**
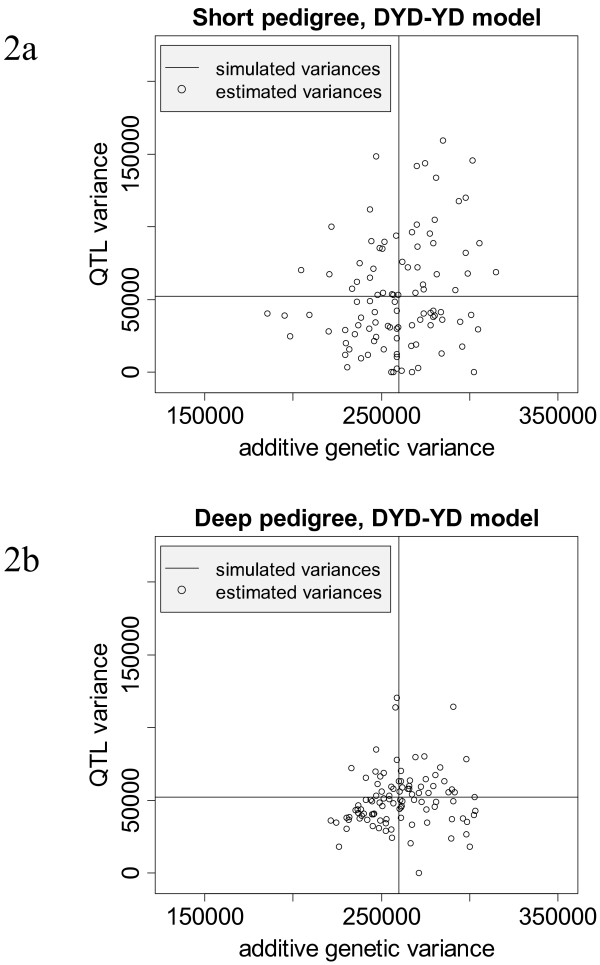
**Estimated (----) additive genetic variance and variance explained by one QTL in comparison to their simulated (----) parameters when phenotypic information is used for bulls and cows**. Phenotypic information corresponds to daughter yield deviations (DYD) of bulls and yield deviations (YD) of cows; Figure 2a presents results for MA-BLUP models with a short depth of pedigrees (see text for details), whereas in Figure 2b deep pedigrees were used for MA-BLUP evaluations.

The effect of increasing the depth of the pedigree is summarized in the second and third lines of Table 4. The values of the estimated components are nearly the same whether the short or deep pedigree is applied, but standard errors indicate an increased accuracy for the estimates in the deep pedigree. The graphs in Figure 2 visualize the estimated standard errors. Increasing the pedigree depth reduces notably the variation between the replicates, which can also be seen from the higher LRT.

Results for the impact of missing genotypes are shown in lines three to five of Table 4. Missing genotypes do not lead to biased estimates for variance components. However, they increase the standard error for ${\hat{\sigma }}_{qtl}^{2}$ whereas the standard errors for all other variance components are hardly affected (see Figure 2).

### Accuracy of MA-BLUP EBV

To evaluate the consequences of the varying conditions for the estimation of MA-BLUP breeding values, accuracies were calculated for proven bulls, cows and young bull candidates. Correlations between simulated and estimated breeding values were assessed for the overall MA-BLUP EBV (2), the residual polygenic breeding value (${\hat{u}}_{i}$ in (2)), and the breeding value at the QTL (QTL-EBV, ${\hat{v}}_{i}$ in (2)). The results shown in Table 5 are again averages of 100 replicates.

**Table 5:** Accuracies of estimated breeding values in MA-BLUP evaluation models that were only based on daughter yield deviations (DYD) for bulls or on DYD for bulls and yield deviations for cows

Phenotypic information	Pedigree depth	Missing genotypes	Proven bulls	Cows	Young bull candidates		
			
			MA-BLUP	MA-BLUP	MA-BLUP	Residual polygenic	QTL-EBV
DYD	short	none	0.942	0.388	0.480	0.437	0.296
DYD-YD	short	none	0.944	0.679	0.556	0.497	0.348
DYD-YD	deep	none	0.945	0.689	0.566	0.501	0.437
DYD-YD	deep	moderate	0.945	0.689	0.563	0.501	0.418
DYD-YD	Deep	extensive	0.945	0.688	0.560	0.500	0.390

Different pedigree structures (short and deep) and levels of missing genotypes (none, moderate and extensive) were applied; accuracies are shown for the overall breeding values of MA-BLUP evaluations, the residual polygenic breeding value and breeding value for the QTL position (QTL-EBV); in breeding value estimation, the estimated variance components were used; the results are averages over 100 replicates per scenario.

Accuracies in AM evaluations were 0.950 for proven bulls, 0.760 for cows and 0.560 for young bulls. Average MA-BLUP accuracies for all categories of animals are hardly affected by the model, pedigree depth or missing genotypes, but strongly affected by the inclusion of YD. The increase in pedigree depth causes a slight improvement of accuracy for young bulls, because the gametic effects can be estimated more accurately. However, this slight increase is gradually lost as the amount of missing genotypes increases.

## Discussion

Questions examined in this study came up during the implementation of MAS for Simmental cattle in Germany and Austria. Our main interest was to find out how to set up an appropriate MA-BLUP system given that it was already decided to use MAS, and in view of the fact that not all relevant animals can be genotyped. In this study, we have investigated the impact of three factors on the estimation of variance components for marker-assisted selection under realistic assumptions: phenotypic information, depth of the pedigree and missing genotypes.

### Design and simulation

Early studies about properties of MAS are in most cases based on nucleus breeding programs [17,18], but this assumption does not hold for the majority of cattle breeding programs in Europe. Therefore, in this study, MAS was integrated in an existing breeding program using a two-step approach as is the case for practical applications in France and Germany [19,7].

Genetic parameters, information content of genetic markers and variance explained by the QTL in the simulation were in agreement with recent results found in German Simmental cattle (unpublished data). Allele frequencies of 0.5 for alternative QTL alleles were assumed to reduce the risk of loosing alternative alleles due to random drift along the simulation process. As mentioned by Rönnegård and Carlborg [20] simulations have shown that random QTL models are capable of giving unbiased estimates even when the QTL is biallelic. Furthermore, the assumptions of a biallelic QTL and balanced allele frequencies reflect findings for QTL in cattle. A well-known example for a biallelic QTL is the K232A substitution in DGAT1. Estimated allele frequencies for alleles at K232A were 0.548 and 0.452 in German Holsteins [21]. Another biallelic QTL is reported for the bovine prolactin receptor in Finnish Ayrshire [22] with allele frequencies of 0.45/0.55 for snp 6. Guillaume *et al*. [23] have reported the proportions of genetic variance explained by single QTL used for the French MAS-program, which range from 5 to 40% for individual QTL. Hence, a QTL explaining 20% of the genetic variance in this study is in the scope of findings for MAS programs in reality. In contrast to practical applications of MAS using multiple QTL [23,7] this study was restricted to a single QTL. Our intention was to examine the influence of different structures of information (pedigree depth, completeness of genotyping) on the estimation of QTL effects. We did not want to examine the effect of several QTL on the accuracy of genetic parameters or on the benefits of MAS. We think that our conclusions will also hold for situations with more QTL, because, in practice, we observe that animals are either genotyped for all QTL or not genotyped at all.

### Choice of phenotypic information

According to our results, the choice of phenotypic information in MA-BLUP models is important. We have shown (Neuner *et al*., [3]) that the two-step approach intrinsically causes a loss of information because not all relatives contribute to the MA-BLUP breeding value. In consequence, some proportion of the QTL information is required to compensate this loss of information. The inclusion of yield deviations for dams reduces this loss of information. As we have shown here, yield deviations also improve the precision for the estimated variance components and deviations from the simulated parameters become smaller. Neuner *et al*. [3] have already discussed the topic concerning the 'choice of weighting factors in MA-BLUP models'. According to the results, the weighting factors daughter equivalents and effective daughter contributions do not introduce bias. One challenge is to combine correctly DYD and YD in one model as these two types of information contain different amounts of genetic and residual variance [3].

### Pedigree depth

The advantage of a more extensive pedigree of genotyped animals for MA-BLUP is obvious. The more animals have complete information, the smaller is the observed standard error of the estimated parameters. Similar to the effect of having more offspring for progeny tested bulls, a deeper pedigree implies more data and more informative matings for the estimation of QTL effects of parents and grandparents in MA-BLUP models. Our results show that using a deep pedigree with many gaps is still preferable over a short but complete pedigree. The reason for this is that a deeper pedigree improves the estimation of polygenic and residual variances as compared to shorter pedigrees.

The effect of a more parsimonious pedigree has also been shown by George *et al*. [13]. By altering the number of offspring per mating from 1.8 to 14.3 offspring per mating, the number of progeny per parent providing information to estimate genetic parameters and MA-BLUP EBV was higher. Even if the approach of George *et al*. [13] was different from the one in our study, similar effects of a larger pedigree were observed: more accurate estimates and increased power.

### Missing genotypes

The third factor investigated was the effect of missing genotypes in MA-BLUP evaluations. If marker information was complete and could be used to infer the transmission of QTL alleles, then the IBD matrix would only contain 1s and 0s. At the other extreme, if no marker information was available, the IBD matrix would become identical to the numerator relationship matrix, *i.e*. all covariance elements with the parents will be equal to 0.5, signifying equal probability of inheriting either allele from a parent. In the end, this would result in identical estimators for residual polygenic and QTL variance. Several approaches exist to deal with the problem that non-genotyped animals do not contribute information for QTL models [13]. A well-known approach is the multiple-site segregation sampler LOKI [14] that was used in this study. We found that missing genotypes did not lead to biased variance components. In contrast to our results, George *et al*. [13] have reported that, if more genotypes are missing, the QTL variance is overestimated, the residual polygenic variance is underestimated and bias is increased. The main reason for these contradictory results could be that the structure of pedigree and missing data in our study allowed a much better reconstruction of missing genotypes by LOKI. In the sheep pedigree of George *et al*. [13] the number of male progeny and grand progeny was smaller than that in the cattle breeding program of this research. Thus, less descendants are available to contribute information for the reconstruction of their ancestors' genotypes. Furthermore, the amount of phenotypic information per sire is very different in both studies. Compared to George *et al*. [13], both the better ability to reconstruct missing genotypes and the higher amount of phenotypic information for MA-BLUP result in unbiased estimates in our study.

### Effects on accuracy of MA-BLUP

A QTL explaining 20% of the additive genetic variance was intentionally chosen, because such a QTL is in the order of magnitude that we observed in Simmental cattle and because it shows nicely that MA-BLUP is not necessarily more accurate than conventional BLUP. Our results show that the accuracy of MA-BLUP breeding values is little affected by the pedigree depth and hardly affected by missing genotypes. However, it is strongly affected by the inclusion of YD in the MA-BLUP system [3]. Guillaume *et al*. [23] have reported empirical results for the accuracies of EBV for young bulls in the French MAS program. The results of our study are in line with their findings for milk yield. When 40% of the genetic variance were marked with four QTL, they found an increase in accuracy for EBV of young bulls in MA-BLUP models of 0.030 compared to the AM model. In our study, we observed accuracies that were 0.006 higher for one QTL explaining 20% of the genetic variance. The main reason for this small increase was the loss of information due to the two-step approach. As mentioned by Neuner *et al*. [3] the loss has to be compensated before additional gain can arise. With respect to accuracy of EBV in MA-BLUP models, Villanueva *et al*. [16] have investigated the benefit of increased pedigree and marker information. They simulated four additional generations of random selection in order to extend their data set. The increased amount of marker genotype information significantly increased the accuracy of the estimation of the QTL effects from 0.54 to 0.65. Parameters in their study were 0.25 for the heritability and 0.24 for the ratio of genetic variance explained by the QTL. Spelman [24] also concluded that if more animals are genotyped in each generation and if more generations of genotypic information are used for MAS, an increase in accuracy of the estimation of QTL effects and therefore in MAS superiority are obtained.

At present many research programs are conducted in order to implement genomic breeding value estimations as described by Meuwissen *et al*. [25]. Although first implementations exist [26] it is not sure that MAS schemes will be abandoned. Improvements in existing MAS programs will directly improve the selection until genomic selection will be applied. We also expect that QTL mapping based on the variance component approach [13] will continue in the future. Our results can be helpful in optimizing the choice of individuals to be genotyped.

In genomic selection projects, which animals to genotype and which source of phenotypic information to use will also have to be decided. Comparable to our study, we expect it will be necessary to rank animals selected for genotyping by their importance in the pedigree and their impact on further generations. For the phenotypic information, applied appropriate weighting and combination of DYD and YD will also be necessary for a correct modeling of genetic and residual variances. As soon as dams will be genotyped for genomic selection, considering their genotypes and YD for parameter estimation will help to improve the accuracy of estimated QTL effects in the same way as in our current study.

## Conclusion

The main conclusion of this study is that phenotypic information of cows and an increased depth of the pedigree have an important impact on the precision of genetic parameters estimated in MA-BLUP models. Furthermore, deep pedigrees with many missing genotypes provide more accurate estimates than short pedigrees with complete genotyping. While the estimation of variance components is considerably improved by a deep pedigree with no missing genotypes, the accuracy of MA-BLUP genetic evaluation is hardly affected by these factors. As a consequence, if exact QTL parameters are available from other sources, the same accuracy of MA-BLUP can also be achieved with a short pedigree and many missing genotypes.

## Competing interests

The authors declare that they have no competing interests.

## Authors' contributions

The questions examined in this study were established from several discussions including all authors. All authors participated in setting up the design, performing the study and helped to draft the manuscript. The main impact of each is as follows: SN wrote the simulation programs, performed the study and wrote the first draft of the manuscript. CE gave support for a realistic modeling of missing genotypes and pedigree depth according to German Simmental cattle, to set up the simulation study using LOKI and for the interpretation of results. RE made suggestions for simulating the population structure, helped during programming and integrating of software packages MIX and ASREML and also checked programs and results. GT participated in setting up the design and conception of the study, helped to discuss the results and to draw conclusions and revised the manuscript to improve readability. KUG conceived the study and helped to perform it, revised large parts of the manuscript with respect to content and readability. All authors read and approved the final manuscript.
